# Diminished mTOR signaling: a common mode of action for endocrine longevity factors

**DOI:** 10.1186/2193-1801-3-735

**Published:** 2014-12-15

**Authors:** Dudley W Lamming

**Affiliations:** Division of Endocrinology, Department of Medicine, University of Wisconsin-Madison, Madison, Wisconsin USA; William S. Middleton Memorial Veterans Hospital, Madison, Wisconsin USA

**Keywords:** Calorie restriction, Rapamycin, Insulin resistance, Longevity

## Abstract

Since the initial observation that a calorie-restricted (CR) diet can extend rodent lifespan, many genetic and pharmaceutical interventions that also extend lifespan in mammals have been discovered. The mechanism by which CR and these other interventions extend lifespan is the subject of significant debate and research. One proposed mechanism is that CR promotes longevity by increasing insulin sensitivity, but recent findings that dissociate longevity and insulin sensitivity cast doubt on this hypothesis. These findings can be reconciled if longevity is promoted not via increased insulin sensitivity, but instead via decreased PI3K/Akt/mTOR pathway signaling. This review presents a unifying hypothesis that explains the lifespan-extending effects of a variety of genetic mutations and pharmaceutical interventions and points towards new molecular pathways which may also be leveraged to promote healthy aging.

**Electronic supplementary material:**

The online version of this article (doi:10.1186/2193-1801-3-735) contains supplementary material, which is available to authorized users.

## Introduction

Calorie restriction (CR), first discovered to extend rat lifespan in the 1930’s, is one of the most effective known techniques for promoting longevity (McCay et al. 1939). A CR diet, in which total caloric intake is reduced while maintaining adequate nutrition, promotes lifespan not only in rats, but in yeast, worms, flies, dogs, and even primates (Lamming and Anderson 2014). A CR diet also increases healthspan – that portion of a life where one is healthy and vigorous. The mechanism underlying the effect of a CR diet on longevity has been hotly debated, with suggestions ranging from a passive mechanism in which CR slows metabolism, to a more active mechanism in which a CR diet induces protective stress response pathways (Anderson and Weindruch 2010; Sinclair 2005).

A conserved response to CR in mammals, including humans as well as non-human primates, is a significant increase in insulin sensitivity (Kemnitz et al. 1994; Cartee et al. 1994). As high-fat, obesity-promoting diets clearly inhibit insulin sensitivity as well as lifespan (Olefsky and Glass 2010), it is logical to suppose that the enhanced insulin sensitivity induced by CR may be responsible for its effects on healthspan and lifespan. In favor of this hypothesis, the Ames and Snell dwarf mice, which have an exceptionally long lifespan, likewise display significantly increased insulin sensitivity (Bartke and Brown-Borg 2004). However, as discussed below, data from genetically modified organisms and the surprising effects of the pro-longevity drug rapamycin on insulin sensitivity show that these effects can be disassociated. The common feature behind many insulin-sensitizing and insulin-desensitizing longevity interventions is decreased mTOR pathway signaling, suggesting that endocrine factors which directly or indirectly regulate mTOR signaling may be potential regulators of longevity.

## Review

### Reduced activity of the PI3K/Akt/mTOR signaling pathway promotes longevity

**Figure 1:**
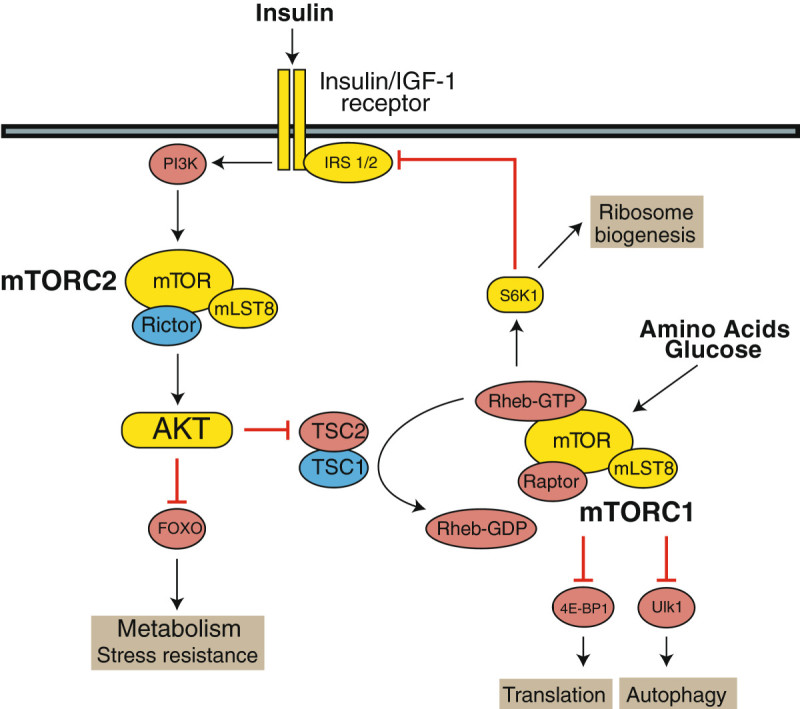
**The PI3K/Akt/mTOR signaling pathway.** Insulin, amino acids and glucose stimulate signaling through the PI3K/Akt/mTOR signaling pathway to regulate ribosomal biogenesis, translation, autophagy, and metabolism and stress resistance.

Reduced signaling through the insulin/IGF-1/mTOR (insulin-like growth factor 1/mechanistic Target Of Rapamycin) signaling pathway has been proposed as an essential mechanism by which a CR diet extends lifespan (Lamming and Anderson 2014). Consistent with this theory, both genetic and pharmacological interventions that reduce signaling through the insulin/IGF-1/mTOR signaling pathway extend lifespan. As detailed in Figure 1, genetic interventions in this pathway reported to extend lifespan include mice null for either *Irs1* or *S6K1*, mice heterozygous for either *Igf1r* or *Akt1*, mice expressing a hypomorphic allele of mTOR, and mice heterozygous for both *mTOR* and *mLST8* (Selman et al. 2009, 2011; Lamming et al. 2012; Bokov et al. 2011; Wu et al. 2013; Nojima et al. 2013). Deletion of the insulin receptor specifically in adipose tissue (the FIRKO mouse) also extends lifespan (Bluher et al. 2003), as does deletion of insulin receptor substrate 2 (IRS2) specifically in the brain (Taguchi et al. 2007).

Although the insulin sensitivity model of CR would predict that all of the interventions noted above should display increased insulin sensitivity, only mice lacking *S6K1* and the FIRKO mouse display increased insulin sensitivity and improved glucose tolerance. Mice heterozygous for *Akt1*, mice expressing a hypomorphic allele of *mTOR*, and mice heterozygous for both *mTOR* and *mLST8* have essentially normal glucose homeostasis. Indeed, mice lacking *Irs1* or that lack *Irs2* specifically in the brain become insulin resistant, while mice heterozygous for *Igf1r* become glucose intolerant and insulin resistant with age (Garg et al. 2011). Mice treated with rapamycin, an FDA-approved immunosuppressive and anti-cancer agent, have a significant increase in lifespan, even when treatment is begun late in life (Harrison et al. 2009). While rapamycin was originally proposed to act as a CR mimetic, analysis of the effects of rapamycin on gene expression have revealed the two interventions to be quite distinct (Fok et al. 2014a). From the standpoint of glucose homeostasis and insulin sensitivity, rapamycin and CR have quite divergent effects, with rapamycin treatment resulting in glucose intolerance and hepatic insulin resistance (Lamming et al. 2012, 2013a). This clearly demonstrates that insulin sensitivity is not required for extended longevity – and that insulin resistance, although perhaps undesirable, is not sufficient to block extended longevity.

It is worth noting that not all genetic interventions in the PI3K/Akt/mTOR signaling pathway that decrease insulin sensitivity promote longevity. For instance, mice heterozygous for expression of the insulin receptor are insulin resistant, but do not have increased mean lifespan (Nelson et al. 2012). Indeed, the ultimate mechanism by which reduced mTOR signaling extends lifespan is unknown. The mTOR protein kinase is found in two distinct complexes (Figure 1), each with distinct functions and substrates. The canonical target of rapamycin is mTOR complex 1 (mTORC1), which is acutely sensitive to rapamycin and regulates ribosomal protein biogenesis, protein translation and autophagy. Extensive genetic studies in yeast and *C. elegans* have demonstrated that inhibition of protein translation or the activation of autophagy is sufficient to extend lifespan (reviewed in (Lamming et al. 2013b)). Deletion of *TSC1* activates mTORC1, resulting in a significant decrease in lifespan due to liver hemangiomas (Kwiatkowski et al. 2002).

While these results suggest that mTORC1 is very important in the response to decreased PI3K/Akt/mTOR signaling, chronic rapamycin treatment also inhibits mTORC2 *in vivo*. Through regulation of key residues on AKT and SGK, mTORC2 regulates the FOXO and p38 MAPK pathways (Lamming et al. 2014). These pathways, in particular the FOXO proteins, are crucial in the regulation of lifespan and stress resistance in *C. elegans*, and siRNA knockdown of *Rictor*, an essential protein subunit of mTORC2, in *C. elegans* extends lifespan. However, male mice heterozygous for *Rictor* have a short lifespan despite normal glucose tolerance. The long life of mice heterozygous for *Akt1* is likely not attributable to activation of FOXO family members, and is instead likely due to decreased mTORC1 activity (Nojima et al. 2013). The role of mTORC2 in the regulation of lifespan is therefore uncertain, and may be mediated largely by its role as a modulator of mTORC1 signaling.

The clear conclusion to be drawn from these studies is that the insulin sensitivity explanation for the positive effects of a CR diet is naïve. Instead, a common theme is that interventions in the PI3K/Akt/mTOR pathway that reduce mTORC1 activity significantly increase lifespan. While much attention has recently focused on rapamycin and genetic mutations in the mTOR complexes and their substrates, at the physiological level mTOR is regulated by a diverse set of physiological stimuli. Some of the most important of these are endocrine factors that collectively serve to coordinate PI3K/Akt/mTOR signaling in multiple tissues. We discuss some of the most important of these pathways below, and highlight opportunities and unanswered questions regarding many of these pathways.

### Insulin-like Growth Factor 1 (IGF-1) signaling and longevity

**Figure 2:**
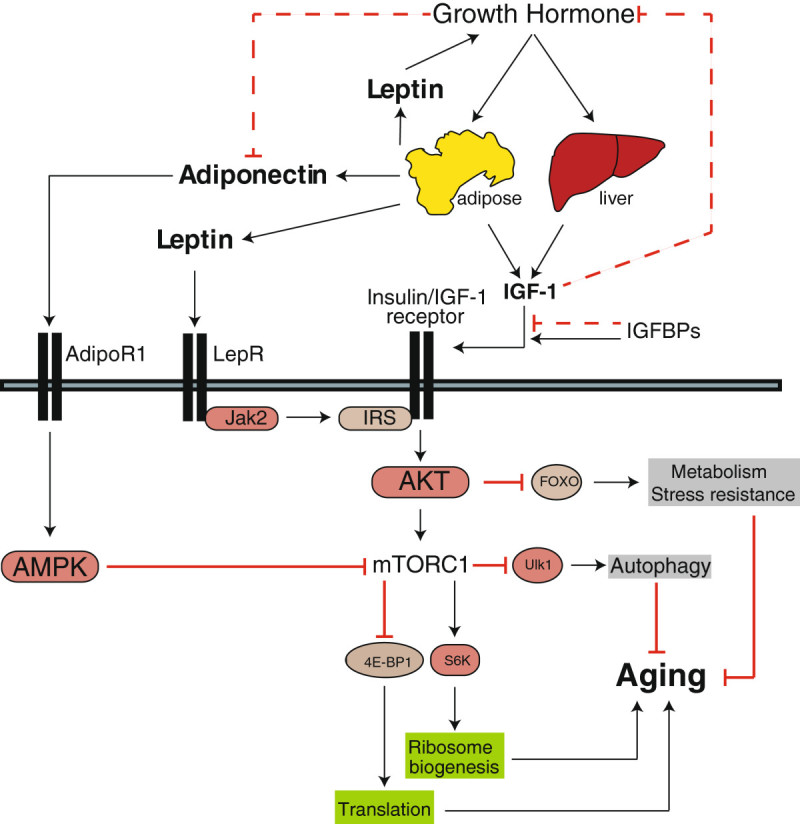
**Regulation of PI3K/Akt/mTOR signaling by growth hormone, IGF-1, leptin and adiponectin.** Growth hormone signaling promotes the expression of IGF-1 by the liver and adipose tissue, which signals through the insulin/IGF-1 receptor to promote PI3K/Akt/mTORC1 signaling and aging. Several mutant mice, such as the Ames Dwarf mouse, are deficient for the production of growth hormone, and consequently have low IGF-1 levels and low mTORC1 activity in IGF-1 sensitive tissues. Growth hormone also normally represses adiponectin, a hormone from white adipose tissue that inhibits mTORC1 activity by activating AMPK. Leptin promotes PI3K/Akt/mTOR signaling via the Jak2-mediated phosphorylation of insulin receptor substrate. Leptin also promotes IGF-1 signaling by stimulating the GH/IGF-1 axis. Functions such as translation and autophagy that impact aging are shown in a green box if stimulated by PI3K/Akt/mTORC1 activity, and gray box if inhibited by PI3K/Akt/mTORC1 activity.

Signaling by insulin and the closely related hormones insulin-like growth factors 1 and 2 (IGF-1 and IGF-2) is mediated by hormone binding to the insulin receptor, the IGF-1 receptor, or hybrid insulin-IGF-1 receptor complexes (van Heemst 2010). Signaling through these receptors activates PI3K/Akt/mTOR signaling and regulates aging (Figure 2). Because of the shared nature of the receptors, which bind each hormone with different affinities, it can be difficult to determine relative contribution of each hormone to metabolic and lifespan effects. Nonetheless, a preponderance of evidence points to a specific role for IGF-1 in the regulation of lifespan in a manner dependent upon PI3K/Akt/mTOR signaling.

IGF-1 is very strongly linked to lifespan, with multiple long-lived mouse models, including the Ames and Snell dwarf mice, the FIRKO mouse, and *Igf1r*^*+/−*^ mice showing decreased levels of IGF-1. Ames and Snell dwarf mice have a primary defect that results in low growth hormone production and therefore extremely low IGF-1 plasma levels (Brown-Borg and Bartke 2012), as well as reduced mTORC1 substrate phosphorylation (Sharp and Bartke 2005). Growth hormone receptor knockout mice have very similar phenotypes in terms of IGF-1 levels, and these mice also live longer. However, a recent publication using mice that produce less IGF-1 shows that while reducing IGF-1 levels in serum and tissue by approximately 50% extends maximum lifespan, it does not increase mean lifespan, suggesting that decreased IGF-1 levels do not fully explain the long lifespan of Ames and Snell dwarf mice (Lorenzini et al. 2014).

IGF-1 does not circulate freely in the blood; instead, circulating IGF-1 is almost always found bound to one of the IGF binding proteins (IGFBPs) which regulate the activity, bioavailability, and retention of IGF-1 (Boisclair et al. 2001). While many contemporary references refer to six IGFBPs, an additional member, IGFBP-7, was characterized almost 20 years ago (Oh et al. 1996). When IGF-1 is bound to either IGFBP-3 and IGFBP-5, a third protein, the acid labile subunit (ALS) is also recruited (Boisclair et al. 2001). The presence of the ALS is critically important in the maintenance of serum levels of IGF-1 and IGFBP3.

While it is clear that IGF-1 activity is regulated by the IGFBPs, this interaction has not yet been thoroughly explored with regard to the regulation of PI3K/Akt/mTOR signaling and lifespan, and individual IGFBPs may promote or inhibit IGF-1 activity (Figure 2). Mice lacking pregnancy-associated plasma protein A, a metalloproteinase that degrades inhibitory IGFBPs and thus increases IGF-1 signaling, have decreased IGF-1 signaling and a significantly extended lifespan (Conover and Bale 2007). With regards to specific IGFBPs, IGFBP2 appears to be the most interesting with regards to the regulation of insulin sensitivity. *Igfbp2* expression is transcriptionally induced in response to leptin in liver and skeletal muscle, and acute overexpression of IGFBP-2 in the liver of *ob/ob* mice significantly improves glucose tolerance and blood glucose and insulin levels (Hedbacker et al. 2010; Yau et al. 2014). While its role on PI3K/Akt/mTOR signaling is unclear, it is actually induced by insulin via the PI3K/Akt/mTOR signaling pathway (Li et al. 2012). However, the effect of IGFBP2 expression on lifespan is not known; indeed, of the IGFBPs, only IGFBP7 has been shown to extend lifespan when overexpressed – in *D. melanogaster* (Alic et al. 2011). Understanding the role of the IGFBPs in mammalian lifespan is likely to prove an important area for future research.

### Growth hormone and Adiponectin regulate PI3K/Akt/mTOR signaling via distinct pathways

The long lifespan of the Ames and Snell dwarf mice, which have a primary defect of impaired growth hormone production, strongly suggest that growth hormone (GH) regulates longevity. Growth hormone Receptor KnockOut (GhRKO) mice, which are unable to sense growth hormone, likewise have extended lifespan. It was discovered in 2005 that Ames dwarf mice have decreased PI3K/Akt/mTOR signaling (Sharp and Bartke 2005), but it has not generally been appreciated that decreased mTOR signaling may be a major mechanism by which decreased levels of GH signaling extend lifespan. As shown in Figure 2, the effect of GH on PI3K/Akt/mTOR signaling is likely mediated in large part by decreased levels of IGF-1.

GH may also promote PI3K/Akt/mTOR signaling through IGF-1 independent pathways. For example, GH has profound effects on methionine metabolism (Brown-Borg and Rakoczy 2013), and amino acids regulate mTORC1 via the Rag family of GTPases (Bar-Peled and Sabatini 2014). GH directly modulates glucose uptake by skeletal muscle (Yakar et al. 2004), and glucose regulates mTORC1 signaling directly via the Rag family of GTPases (Efeyan et al. 2013). Another major effect of GH on physiology is altered lipid metabolism, with Ames dwarf mice showing significantly reduced levels of plasma free fatty acids and tissue triglyceride levels and decreased body fat in adults (Wang et al. 2006; Heiman et al. 2003). Reduced lipid levels could promote insulin sensitivity and thus impact PI3K/Akt/mTOR signaling.

Adiponectin is a protein hormone secreted from white adipose tissue that is heavily implicated in the extension of lifespan, being increased in mice on a CR diet as well as GHRKO and Ames dwarf mice (Wang et al. 2006). It was first discovered as a hormone that is decreased in the serum of humans with type 2 diabetes, and that was increased upon weight loss (Hotta et al. 2000). Adiponectin exerts antidiabetic effects in part by increasing hepatic insulin sensitivity and decreasing skeletal muscle insulin resistance (Yamauchi et al. 2001; Berg et al. 2001). Adiponectin action is meditated by the adiponectin receptors AdipoR1, which activates AMPK, and AdipoR2, which activates the PPARα signaling pathway. The increase in skeletal muscle insulin sensitivity is mediated by the activation of AMPK, which leads to the inhibition of mTORC1 (Figure 2), decreasing the activation of its substrate S6K1 and reducing the inhibitory serine phosphorylation of IRS1 (Wang et al. 2007). There is significant interest in adiponectin as a potential mediator of the beneficial effects of a CR diet, but ongoing studies have not conclusively linked adiponectin levels to longevity in humans (Stenholm et al. 2011).

Overexpression of adiponectin in mice leads to a significant increase in lifespan on both normal and high-fat diets (Otabe et al. 2007). Many of the metabolic effects of adiponectin overexpression are similar to those of Ames dwarf mice and GhRKO mice (Brown-Borg and Bartke 2012). Fascinatingly, decreasing the elevated adiponectin levels of GhRKO mice by surgical removal of the visceral fat depots leads to normalization of metabolic phenotypes associated with longevity, including insulin sensitivity, body temperature, and respiratory quotient (Masternak et al. 2012). It remains to be seen if this surgical intervention also normalizes the lifespan of GhRKO mice; however, it is apparent from genetic experiments that loss of GhR specifically in adipose tissue is not sufficient to increase adiponectin or increase insulin sensitivity (List et al. 2013). Investigation of PI3K/Akt/mTOR signaling in these mouse models of normalized adiponectin expression may provide insight into the possible effects of these interventions on longevity.

One important distinction between the Ames dwarf mouse and the GhRKO mouse is that the long lifespan of the Ames dwarf mouse can be extended by a CR diet – an effect mediated by GH (Gesing et al. 2014) – whereas GhRKO mice are largely insensitive to the effects of a CR diet on lifespan (Bonkowski et al. 2006). Notably, wild type mice placed on a CR diet have increased insulin sensitivity and decreased signaling to Akt, but these effects do not occur in GhRKO mice (Al-Regaiey et al. 2005). While it is not clear why the GH receptor is required for the effect of CR, these results correlate with a model in which CR promotes lifespan via decreased PI3K/Akt/mTOR signaling.

### Leptin promotes PI3K/Akt/mTOR pathway signaling

Understanding the source of hunger and satiety has been an area of significant research in the face of the obesity epidemic sweeping much of the world. Leptin, the most famous of the satiety hormones, is a hormone produced by white adipose tissue. Leptin was identified by positional cloning of the spontaneous mouse *ob* mutation (Zhang et al. 1994), leading to the identification of a small serum protein that regulates energy expenditure and food intake (Halaas et al. 1995). By regulating food intake and obesity, leptin indirectly impacts insulin sensitivity, but emerging evidence suggests that leptin may also directly regulate insulin signaling. Expression of leptin receptor in neurons of the hypothalamic arcuate nucleus improves insulin sensitivity (Morton et al. 2005).

As outlined in Figure 2, it is now known that leptin activates the PI3K/Akt/mTOR signaling pathway via Jak2 mediated phosphorylation of insulin receptor substrate (Park and Ahima 2014). Leptin also promotes PI3K/Akt/mTOR signaling via activation of the GH/IGF-1 axis (Watanobe and Habu 2002). The role of leptin in healthy aging and lifespan has been the subject of significant study. The dramatic phenotype of *ob/ob* and *db/db* mice that are deficient for leptin signaling clearly indicates that intact leptin signaling is required for healthy aging. Leptin resistance increases with age, and it has been suggested that diminished leptin action may be a cause of aging (Gabriely et al. 2002).

Leptin is reduced by a CR regimen (Schlitt and Schulz 2012), and the role of leptin in promoting PI3K/Akt/mTOR activity (Figure 2) would lead us to predict that decreased leptin levels would be beneficial for lifespan. However, despite the positive effect of leptin on PI3K/Akt/mTOR signaling, the net physiological consequence of leptin administration is a reduction in food intake and body weight (Halaas et al. 1997). Transgenic leptin mice have reduced food intake and an approximately 60% decrease in serum insulin levels (Qiu et al. 2001), which is likely to result in a net drop of PI3K/Akt/mTOR activity. The effect of leptin overexpression or lifelong administration of leptin on lifespan has not yet been examined. Leptin is a fascinating case in which the physiological context in which PI3K/Akt/mTOR pathway signaling is regulated may modify the net effect of the hormone on lifespan.

### The role of many other hormones in PI3K/Akt/mTOR and lifespan is not yet clear

While the role of growth hormone, IGF-1, adiponectin, and leptin in the regulation of PI3K/Akt/mTOR signaling is becoming clear, there are many other metabolic regulatory hormones that may also play a role in the regulation of PI3K/Akt/mTOR pathway signaling and longevity. However, significant additional research will be required to elucidate these links. In the following section, we provide a brief introduction to resistin, ghrelin, cholecystokinin (CCK), glucagon like peptide 1 (GLP-1), the fibroblast growth factors, and humanin, all of which impinge upon glucose metabolism and insulin signaling, and are thus also candidate factors which may regulate PI3K/Akt/mTOR activity and thus regulate lifespan.

#### Resistin

Resistin is a protein hormone secreted by white adipose tissue, but in contrast to adiponectin, resistin acts to decrease insulin sensitivity (Steppan et al. 2001). Resistin was initially discovered in a screen for adipocyte genes responsive to treatment with the anti-diabetes drug rosiglitazone, and blocking the action of resistin with an antibody significantly increases glucose tolerance in mice. Resistin levels are decreased by a CR regimen in rats, likely mediated by GH and IGF-1 (Chiba et al. 2008), and are also decreased in the fat of GhRKO mice (Masternak et al. 2012). While longevity studies have not been performed with mice overexpressing or lacking resistin, a recent paper found that resistin impacts cholesterol metabolism by regulating the expression of low-density lipoprotein receptor (Melone et al. 2012), suggesting an important role for this hormone in healthspan.

#### Ghrelin

Ghrelin, the so-called “hunger hormone”, is released primarily by endocrine cells in the stomach when the stomach is empty. Ghrelin acts as a neuropeptide that effectively antagonizes the action of leptin, and many of the same cells in the arcuate nucleus of the hypothalamus express both leptin and ghrelin receptors. However, ghrelin receptors are also expressed in other tissues (Geary 2004). Deletion of the receptor for ghrelin, the growth hormone secretagogue receptor, significantly improves insulin sensitivity during aging via the regulation of fat metabolism (Lin et al. 2011). Administration of ghrelin has some positive effects on the physiology of middle aged mice, reducing body weight primarily via a reduction in fat mass (Ariyasu et al. 2008). While no true lifespan study has been performed in healthy mice, it has been speculated that ghrelin might be an “anti-aging hormone” (Maejima et al. 2011). However, a recent study has linked ghrelin signaling in the hypothalamus to the activation of mTORC1 (Stevanovic et al. 2013), which would lead us to predict (Figure 2) that ghrelin might actually promote aging. As with leptin, it is likely that thoroughly understanding the physiological context in which ghrelin regulates PI3K/Akt/mTOR signaling will be required to understand the role of ghrelin in longevity.

#### Cholecystokinin

Cholecystokinin (CCK) is a hormone released by duodenal I-cells that stimulate gallbladder contraction and pancreatic exocrine secretion, but it is also a neuropeptide that modulates satiety (Lavine et al. 2010; Dufresne et al. 2006). CCK receptor antagonists and agonists have attracted pharmaceutical company attention as a means of modulating feeding behavior, as well as treating pain and anxiety. With the exception of proglumide, a drug that inhibits gastric secretions and is used in ulcer treatment (Bergemann et al. 1981) CCK-based therapies have not successfully translated to the clinic. For example, the CCK agonist GI181771X was tested in clinical trials as a treatment for obesity, but humans taking GI181771X did not lose weight (Jordan et al. 2008).

CCK is actually a family of related peptides produced by processing of pre-pro-cholecystokinin, and are also often sulfated – in fact, sulfated CCK-8 is the most bioactive form. It has been known since the 1980’s that in addition to their role in satiety, CCK also plays a role in the regulation of glucose homeostasis, and CCK-8 stimulates insulin secretion in both rodents and humans (Ahren et al. 2000). CCK may also play an important role in the regulation of beta cell mass (Lavine et al. 2010; Linnemann et al. 2014). In pancreatic acinar cells, CCK promotes PI3K/Akt/mTOR signaling (Williams et al. 2002). OLETF rats, which have a spontaneously arising mutation in the CCK-A receptor, develop late onset hyperglycemia as well as diabetic nephropathy. OLETF rats have decreased cardiac IGF-1 expression as well as decreased phosphorylation of cardiac Akt (Makino et al. 2009). Although no longevity studies have been conducted with exogenously delivered CCK, it is therefore likely that CCK activates PI3K/Akt/mTOR signaling. However, CCK also acts synergistically with leptin to promote satiety, possibly by activating AMPK in the hypothalamus (Akieda-Asai et al. 2014), which may inhibit mTORC1 signaling. It therefore appears likely that the context in which CCK activation takes place may be important in understanding its effect on PI3K/Akt/mTOR activity.

#### Glucagon-like peptide 1 (GLP-1)

Glucagon-like peptide 1 (GLP-1) is a hormone derived from processing of the proglucagon gene product by the small intestine in response to nutrients. GLP-1 potently stimulates insulin secretion, but its activity is short-lived due to the action of dipeptidyl peptidase-4 (DPP4), a circulating enzyme which cleaves and inactivates GLP-1 (Kimple et al. 2014). Several drugs based on GLP-1 are now approved, including exenatide and liraglutide, GLP-1 agonists that are resistant to cleavage by DPP4, and sitagliptin, an inhibitor of DPP4.

GLP-1 has shown success in reversing some of the phenotypes of aging, specifically in reversing the age-related decline in glucose tolerance in aged rats (Wang et al. 1997). This effect is due in part to the effects of GLP-1 on beta cell function. GLP-1 administration increases insulin content and potentiates glucose stimulated insulin secretion in aged rats, while also stimulating increased beta cell mass and proliferation (Doyle and Egan 2001). Although no aging studies have been conducted with GLP-1 in healthy aged animals, GLP-1 has attracted some excitement as a possible therapy for diseases of neuronal injury, including Alzheimer’s disease, stroke, Huntington’s disease, and Parkinson’s disease (Li et al. 2009, 2010; Martin et al. 2009). Indeed, two clinical trials of the GLP-1 agonist liraglutide in Alzheimer’s disease are in progress; no results have yet been reported. Perhaps surprisingly for a drug with so many positive effects in neuronal injury models, liraglutide activates PI3K/Akt/mTORC1 signaling in beta cells (Miao et al. 2013). Understanding the mechanism by which GLP-1 agonists may promote healthspan and longevity is clearly a ripe area for future research.

#### Fibroblast growth factors

The role of fibroblast growth factors in aging was an unexpected discovery, arising from investigations into the ability of the *Klotho* gene to suppress aging (Kuro-o et al. 1997; Kurosu et al. 2005). It was noticed that the phenotypes of *Klotho* mutant mice - which include infertility, kyphosis, atherosclerosis, skin atrophy, muscle atrophy, T-cell dysregulation, pulmonary emphysema, altered phosphate and calcium metabolism, and shortened lifespan – are similar to the phenotype of mice lacking *Fgf23* (Kurosu and Kuro 2009). This suggested that Klotho and FGF23 might function in the same pathway, and it was soon realized that Klotho and its homologue βKlotho form complexes with FGF receptors, increasing the affinity of the receptor for FGF23 in the case of Klotho, and FGF15/19 and FGF21 in the case of βKlotho (Kurosu and Kuro 2009). Although initially unclear, it is now believed that FGF23 does not play a vitamin D – independent role in either glucose homeostasis or aging (Streicher et al. 2012).

In contrast, FGF21 is well-established as an insulin-sensitizing hormone produced in the liver in response to fasting or a CR diet (Kuhla et al. 2014). FGF21 antagonizes the GH-IGF1 signaling axis by in the liver by blocking Jak2/Stat5 signaling and suppressing the transcription of *Igf1* while also inducing *Igfbp1* (Inagaki et al. 2008). Mice that overexpress FGF21 have an approximately 30% increase in male lifespan, with female lifespan being extended by over 40% (Zhang et al. 2012). They also have significantly improved glucose homeostasis, with lower fasting glucose and insulin levels, and improved whole body insulin sensitivity (Zhang et al. 2012). Surprisingly, FGF21 expression is positively mediated by PI3K/Akt signaling (Izumiya et al. 2008), and its expression does not increase during CR (Mendelsohn and Larrick 2012). Although FGF21 may be a partial CR mimetic, it likely functions via a distinct mechanism from the PI3K/Akt/mTORC1 signaling discussed above.

#### Humanin

Humanin is a short, 24-residue peptide originally identified as the result of a cDNA library screen for neuroprotective genes (Tajima et al. 2002). It was immediately noted that the open reading frame for humanin was identical to that of mitochondrial 16S ribosomal RNA, resulting into some controversy as to whether the protein for humanin was expressed. Interestingly, although humanin binds to IGFBP-3 both *in vitro* and *in vivo* (Ikonen et al. 2003), research on the role of humanin remained focused on its potential role as a neuroprotective agent. However, as researchers began to explore the potential role of insulin resistance in the pathogenesis of Alzheimer’s disease, it was found that humanin is also a central regulator of peripherial insulin sensitivity (Muzumdar et al. 2009). Humanin also promotes beta cell survival in nonobsese diabetic (NOD) mice, delaying the onset of diabetes and improving glucose tolerance (Hoang et al. 2010). The actions of humanin on glucose homeostasis are likely mediated in part by its promotion of glucose stimulated insulin secretion by beta cells (Kuliawat et al. 2013).

While the effect of humanin on lifespan has not yet been determined, humanin and humanin analogues have shown efficacy in mouse models of cardiac injury, as well as positive effects on neuronal survival and memory in the mouse models of Alzheimer’s disease, suggesting that humanin treatment may lead to increased longevity (Tajima et al. 2005; Muzumdar et al. 2010). It will be interesting to learn if humanin regulates PI3K/Akt/mTOR signaling, either directly or via modulation of IGF-1. Future study of humanin and other mitochondrial derived peptides are likely to lead to important biological discoveries in the biology of aging.

## Conclusions

Herein, we have discussed some of the major endocrine factors that regulate glucose homeostasis and their effects – or possible effects – on longevity. We have presented a unifying model in which growth hormone, IGF-1, adiponectin, and leptin may all regulate lifespan via their effect on the PI3K/Akt/mTOR signaling pathway. While it remains to be conclusively proven, a CR diet may similarly act via decreased PI3K/Akt/mTOR signaling. Decreased fasting blood glucose and insulin levels are widely conserved effects of a CR diet in mammals (Lamming and Anderson 2014), and a CR diet significantly inhibits the PI3K/AKT/mTOR signaling pathway even in humans (Heilbronn et al. 2006; Mercken et al. 2013). At least some genetic mouse models of increased insulin sensitivity have a short lifespan (Nelson et al. 2012), again correlating with a model in which increased PI3K/AKT/mTOR signaling pathway activity acts to limit lifespan.

We have also briefly touched on a number of other endocrine factors that may regulate longevity via this same PI3K/Akt/mTOR signaling pathway. While some of these factors have established connections to PI3K/Akt/mTOR signaling, future research will be required to learn if the others regulate this pathway. There are many other insulin sensitizing hormones we have not discussed, including prolactin, which regulates hepatic insulin sensitivity (Yu et al. 2013) as well as beta cell function and mass (Park et al. 2012); the recently discovered meteorin-like which regulates beige fat thermogenesis (Rao et al. 2014); and the lipocalin family of hormones, which includes LCN2 (Rao et al. 2014), LCN13 (Zhou and Rui 2013), and Mup1 (Major urinary protein 1) (Zhou et al. 2009). While the role of these proteins in the regulation of lifespan and the PI3K/Akt/mTOR signaling pathway is largely unknown, this area is ripe for study.

**Figure 3:**
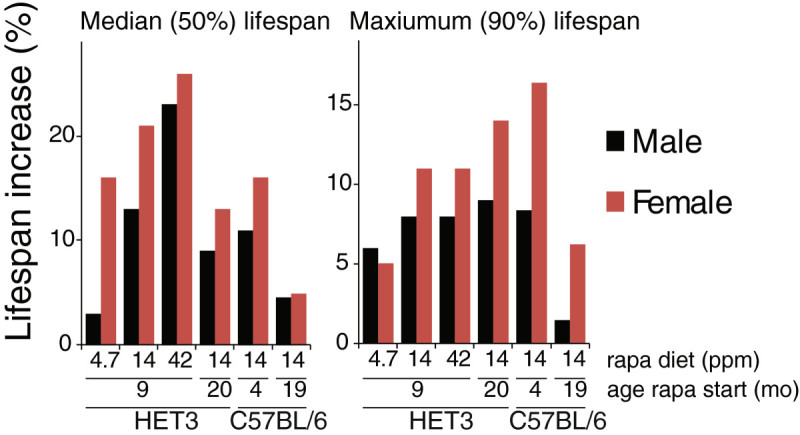
**The sexually dimorphic effect of rapamycin treatment.** Rapamycin consistently has a stronger effect on average and maximum female lifespan than on male lifespan in both HET3 and C57BL/6 mice. Data taken from (Zhang et al. 2014; Miller et al. 2014; Harrison et al. 2009; Fok et al. 2014b); median lifespan not available for HET3 mice initiated on 14ppm rapamycin at 20 months of age, mean is shown instead.

Finally, we have discussed that the context in which PI3K/Akt/mTOR signaling is regulated may be critical to understanding the effect on longevity. One of the most significant contexts is sex, and both genetic and pharmaceutical interventions in the insulin/IGF-1/mTOR signaling pathway consistently show greater benefits in females than males. This sexual disparity in lifespan extension is observed in mice null for either *Irs1* or *S6K1* (Selman et al. 2009, 2011), mice heterozygous for both *mTOR* and *mLST8* (Lamming et al. 2012), and consistently and across a range of doses in mice treated with rapamycin (Figure 3) (Miller et al. 2014). While the mechanistic and physiologic basis for this effect is unknown, 17β-estradiol plays a protective role against the development of rapamycin-induced diabetes, suggesting a possible contribution of sex hormones (Schindler et al. 2014). A deeper understanding the role of sex hormones and other endocrine factors in the PI3K/Akt/mTOR-dependent regulation of longevity will provide a platform for the development of interventions that can extend lifespan across the sexes and in a wide range of physiological contexts.

## Authors’ original submitted files for images

Below are the links to the authors’ original submitted files for images. Authors’ original file for figure 1 Authors’ original file for figure 2 Authors’ original file for figure 3

## Abbreviations

CCK: Cholecystokinin
Fgf21: Fibroblast growth factor 21
GhRKO: Growth hormone receptor knockout
GLP-1: Glucagon-like peptide 1
IGF-1: Insulin-like growth factor 1
mTOR: Mechanistic target of rapamycin.
