# Supplementary figures and images for: Diminished mTOR signaling: a common mode of action for endocrine longevity factors
Source: Springerplus. 2014 Dec 15;3:735. doi: 10.1186/2193-1801-3-735 (PMC4320218; doi:10.1186/2193-1801-3-735)

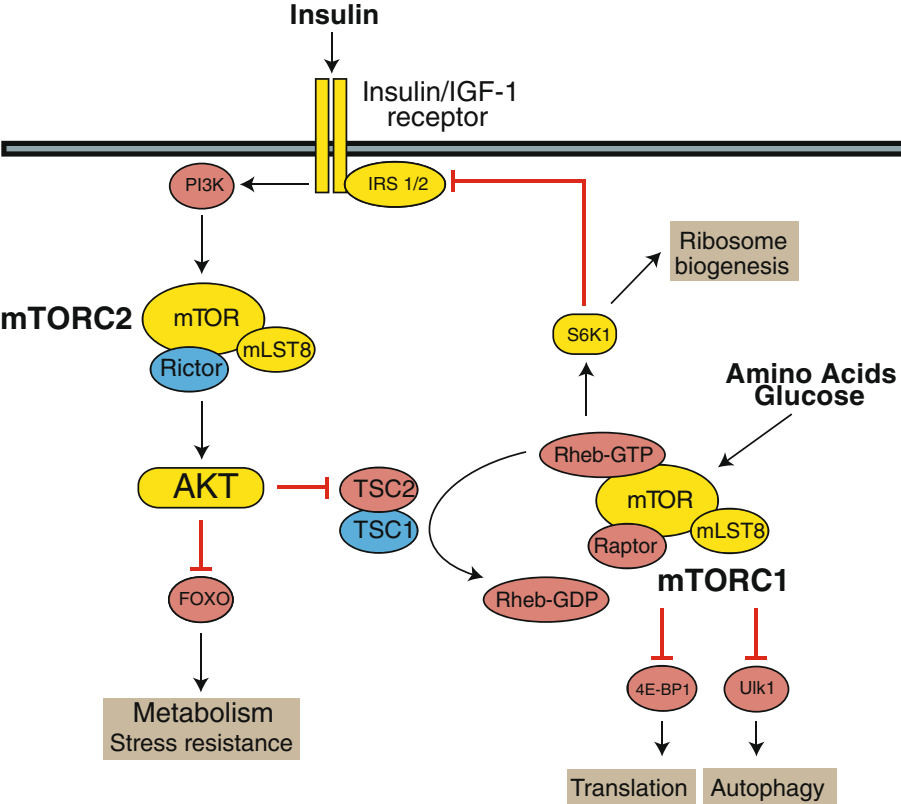

Supplement: Supplementary file 1 — Authors’ original file for figure 1 [file 40064_2014_1498_MOESM1_ESM.pdf]

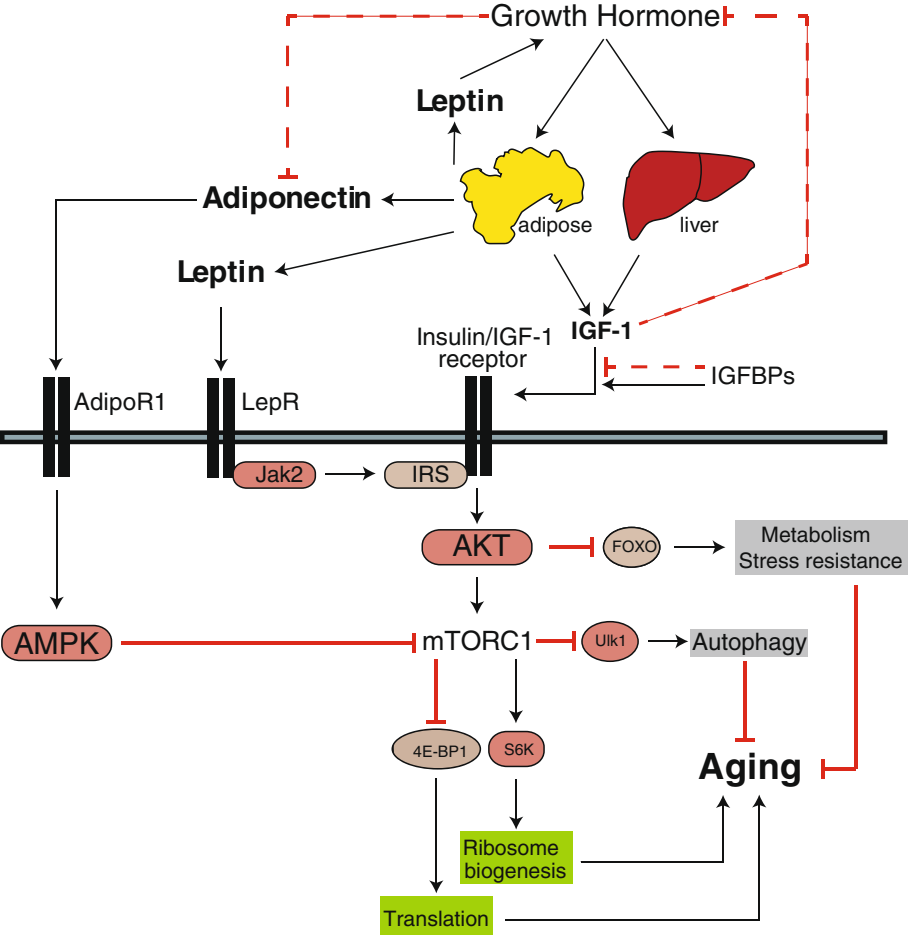

Supplement: Supplementary file 2 — Authors’ original file for figure 2 [file 40064_2014_1498_MOESM2_ESM.pdf]

Lifespan increase (%)

Median (50%) lifespan

Maximum (90%) lifespan

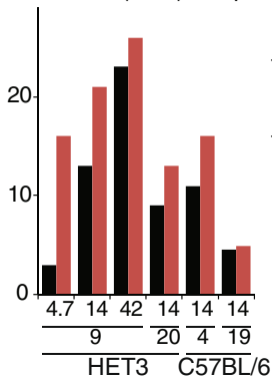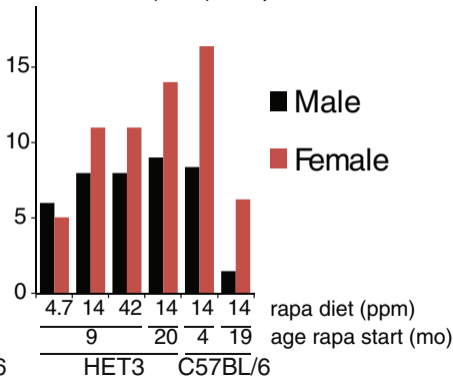

Supplement: Supplementary file 3 — Authors’ original file for figure 3 [file 40064_2014_1498_MOESM3_ESM.pdf]
